# The hunt for red October (or November)

**DOI:** 10.3325/cmj.2021.62.300

**Published:** 2021-06

**Authors:** Charles H. Calisher

If you have seen it, I am sure that you will agree with me that “The Hunt for Red October” is a good movie. Fiction, of course, but very convincing. Good actors (particularly Sean Connery), good dialogue, much action, not much scenery but what is there is adequate and technically realistic and plausible, danger is averted. At the present time the world has a real situation on its hands, one far more dangerous than a movie. At the time of this writing, the current pandemic, caused by severe acute respiratory syndrome coronavirus virus type 2 (SARS-CoV-2), has caused more than 168 million cases, including 3.5 million deaths (sources: Wikipedia, The New York Times, and Johns Hopkins University Center for Systems Science and Engineering). At this same moment, the virus is ravaging India (having infected 27.2 million people, including 311 000 dead; obvious underestimations), and elsewhere in Asia, Europe, Africa, Europe, Oceania, and North and South Americas. A few cases have occurred among the few thousand people in Antarctica. The COVID-19 human, and perhaps zoonotic, pandemic is understandably considered the most severe of our lifetimes. Accompanying individual disasters have been economic hardships, personal and population mental health traumas, reduced educational opportunities, childhood distress, broken family bonds, reduced business income, world economic effects, increased human trafficking, impacts on climate changes, food shortages, increased livestock diseases, domestic violence, and human population decreases, not to speak of increased costs to manage governments and non-governmental organizations.

In 2002, an outbreak of a β-coronavirus eventually named severe acute respiratory syndrome coronavirus virus type 1 (SARS-CoV-1) occurred in China and was followed by a limited global spread. As often happens after an outbreak or epidemic, that “disease of the week,” as some euphemistically called them, emphasis and accompanying necessary funding diminished when SARS-CoV-1 essentially disappeared after the initial outbreak. Nonetheless, in 2007 scientists studying coronaviruses warned that “The presence of a large reservoir of SARS-CoV–like viruses in horseshoe bats [genus *Rhinolophus*] is a time bomb. The possibility of the re-emergence of SARS and other novel viruses should not be ignored.” (1).

In 2012, another previously unknown β-coronavirus, this one named Middle East respiratory syndrome coronavirus (MERS-CoV), and closely related to SARS-CoV-1, emerged to cause high case-fatality human infections. Fortunately, this virus is not transmitted efficiently between humans, and cases have been largely limited to the Middle East where its intermediary host, the dromedary camel (*Camelus dromedarius*), is present in relatively high numbers. Then, in 2016, yet another novel bat-origin coronavirus, an α-coronavirus, emerged in China to cause a novel epizootic disease in pigs, termed swine acute diarrhea syndrome coronavirus (SADS-CoV).

Now, 19 years since SARS-CoV-1 was recognized, COVID-19 has emerged as the deadliest respiratory disease pandemic since the influenza pandemic killed an estimated 50 million people in 1918 (2). Clearly, we need to understand the natural history of SARS-CoV-2 so that we can prevent a similar occurrence in the future and be a great deal more prepared to contain coronavirus and other viruses with pandemic potential at their outsets, as has been suggested previously (3).

## CAN WE UNDERSTAND THE PANDEMIC EMERGENCE OF THESE VIRUSES BY FOCUSING ON THE EMERGENCE OF SARS-COV-2?

Prior to the recognition of SARS-CoV-1 in 2002, the genetic sequences of that virus had not been identified in viruses of humans or animals. This virus has not been detected since then, likely due to an aggressive public health response in China and elsewhere. Scientific research conducted over the past two decades provides clues about how and why the COVID-19 pandemic appeared. We must understand these critically important scientific findings, so that we can better address significant risks we likely will continue to face for the foreseeable future. Sean Connery died a year ago, so we will have to do this on our own, without his dramatic heroics.

## FIRST NEED, A VACCINE OR VACCINES

These usually take years to develop and be approved by national vaccine advisory groups. The process typically is meticulous and lengthy. However, and in view of this pandemic emergency, various commercial and national laboratories have risen to the occasion and produced novel vaccines that are efficient and meet the approval of the relevant authorities. Appropriate distribution and use of these vaccines is a separate issue and will not be discussed further here.

## WHERE WAS THE INDEX CASE AND WHEN DID IT OCCUR?

This is THE key issue. However, information needed to answer these questions has been so distorted by self-protecting governmental representatives and by the media that discussion of this issue has become little more than a jumble of reports conflating the opinions of reputable scientists with accusations, counter-accusations, denials, and obvious political allegations by others. It is difficult for anyone to determine what the facts of this matter actually are.

Last year, I was asked to sign on to a manuscript to be submitted to *The Lancet*, an elegant and influential journal. To summarize, the manuscript indicated that the authors believed that accusations that the then-epidemic of SARS-CoV-2 resulted from an act on the part of the Chinese government were unsubstantiated. We indicated that we believed there was not enough data, one way or the other, to make any such statement and that the world should wait until there was such information available. The person (author) who drafted the manuscript listed the authors (many outstanding scientists, most of whom I know) alphabetically and since then my computer has not stopped bringing me requests from people world-wide who have taken exception to some of what we published (4). These people all want to know whether I really believe what we said and ask whether I would like to withdraw my statements in light of recent revelations and supposed revelations (ie, rumors, innuendos, lies, propaganda, etc). I still believe we were correct, insofar as there is still no definitive information regarding the source of this virus.

## NOW WHAT?

Recent investigations by numerous scientific and media personnel have provided conflicting reports about people in China, some of whom had died or been hospitalized or just were unwell with a COVID-19-compatible illness before the first cases of this illness were officially reported in late 2019. One Chinese graduate student mentioned in a publication (later withdrawn) that serum samples from some of these patients had indicated infections with SARS-CoV-2.

Without going into the endless and mostly un-useful details about all this, here is my summary, not at all to be taken as definitive: Miners harvesting bat guano, which can be used as fertilizer, were shoveling this guano in a mine at Beng-ping in Mojiang county, Yunnan Province in 2012. Six of these miners fell ill with signs and symptoms of COVID-19 and three of them died. Researchers from the Wuhan Institute of Virology traveled to the mine in 2013 and returned with samples taken there from bats. According to the Chinese government, the samples they collected yielded no evidence of the presence of a coronavirus closely related to SARS-CoV-2. What caused these miners’ illnesses and deaths remains unanswered. The team of investigators sent by W.H.O. to Wuhan wrote a report on their investigations but their investigations were incomplete because the Chinese government did not allow them to see all the laboratory’s files.

Other discussions and comments from far and wide have centered on various and quite differing possibilities in regard to where this virus originated and how it burst forth as one case, then a cluster of cases, then an epidemic, and now a pandemic. Unless and until the Chinese government becomes suddenly transparent and allows international investigators to review all the files in Wuhan and elsewhere, we will not know the origin of this virus and we may never know it. If the first human infection with SARS-CoV-2 occurred inadvertently (no one is perfect) and then escaped detection to start all this mess, we will all be greatly relieved. We know that as many as half of all infections with this virus are asymptomatic, so this brings us to the real possibility of an unrecognized infection in an individual who had worked in those laboratories in Wuhan.

Among the plethora of publicly published articles, scientific papers, television and radio reports, letters to editors, and so on, I recommend one, by Nicholas Wade, that carefully, and in detail, compares what can be called “data” and common sense (which is not always “common” and does not always make “sense”) and which draws plausible conclusions (5). Wade says, “Neither the natural emergence nor the lab escape hypothesis can yet be ruled out. There is still no direct evidence for either. So, no definitive conclusion can be reached.” Nonetheless, Wade’s logic draws one toward plausible conclusions and makes for informed reading.

I suggest that no one stay up nights wondering how this all began, although knowing how it all began is something we need. If there are data available or potentially available, keeping its existence secret is a huge disservice to us all. Fall-out from even the appearance of a conspiracy theory might be enough to twist good relations into bad and dangerous ones.
